# Brownfield land and health: A systematic review of the literature

**DOI:** 10.1371/journal.pone.0289470

**Published:** 2023-08-04

**Authors:** Weiyi Wang, Sarah Dack, Ian Mudway, Holly Walder, Bethan Davies, Robie Kamanyire, Daniela Fecht

**Affiliations:** 1 UK Small Area Health Statistics Unit, MRC Centre for Environment and Health, School of Public Health, Imperial College London, London, United Kingdom; 2 National Institute for Health and Care Research Health Protection Research Unit in Chemical and Radiation Threats and Hazards, School of Public Health, Imperial College London, London, United Kingdom; 3 UK Health Security Agency, London, United Kingdom; 4 MRC Centre for Environment and Health, Environmental Research Group, School of Public Health, Imperial College London, London, United Kingdom; United States Environmental Protection Agency, UNITED STATES

## Abstract

**Background:**

Brownfield land is vacant or derelict land that was previously used for industrial or commercial purposes. Brownfield land is increasingly being targeted for housing development, however, depending on the previous use and remediation activity, it might pose potential risks to the health of residents on or in the vicinity of redeveloped sites. This systematic review of the literature synthesises the empirical evidence on the associations between brownfield land and health.

**Methods:**

We systematically searched EMBASE, MEDLINE, Global Health, Web of Science, Scopus and GreenFile using a study protocol registered on PROSPERO (CRD42022286826). The search strategy combined the keywords “brownfield” and its interchangeable terms such as “previously developed land”, and any health outcomes such as “respiratory diseases” and “mortality”. Publications identified from the search were screened for eligibility by two authors, and data were extracted from the selected articles. Study quality was assessed based on the Newcastle-Ottawa Scale.

**Results:**

Of the 1,987 records retrieved, 6 studies met the inclusion criteria; 3 ecological studies, 2 cross-sectional studies, and 1 longitudinal study. There was considerable heterogeneity in the exposure metrics and health outcomes assessed. All studies found significant positive associations between brownfield land proximity or density with at least one health relevant outcome, including poorer self-reported general health, increased mortality rates, increased birth defects, increased serum metal levels, and accelerated immune ageing.

**Conclusions:**

Brownfield land may negatively affect the health of nearby residents. The epidemiological evidence on health effects associated with brownfield land in local communities, however, remains inconclusive and limited. Further studies are required to build the evidence base to inform future housing policies and urban planning.

## Introduction

The increasing urban population [1] has put significant pressure and demand on the available land for urban housing development. The overuse of greenfield land–undeveloped green land, generally located on the suburban fringes–for housing development has raised important environmental concerns, as the land is very likely to lose its ability to provide ecosystem services for nature and the climate [2]. In contrast, brownfield land is previously used industrial or commercial land that is now vacant or derelict. Brownfield land is mostly located in cities and urban areas where infrastructure such as roads, water and electricity supply, sewerage and schools are already in place. Therefore, the reuse of brownfield land for residential or commercial development is economically viable and environmentally sustainable [3]. The redevelopment of brownfield land for housing has been promoted in many countries. The U.S. Environmental Protection Agency (EPA), for example, established the “Brownfield Economic Redevelopment Initiative” in the early 1990s to offer funds for local governments to assess brownfield land. The U.S. government also provided financial incentives, such as tax cuts, to encourage the clean-up and redevelopment of brownfield land [3]. In the UK, the Brownfield Land Registers requires local planning authorities to publish up-to-date and publicly available information on brownfield land that is considered suitable for residential development, aiming to encourage new housing on brownfield land [4]. Following that, the government has released funds for local councils to transform brownfield land to new homes [5].

Due to its broad definition, brownfield land could previously have a wide range of uses, but predominant uses include industrial activities such as manufacturing, processing, storage facility, petrol station and oil plant [3,6]. Some of these previous uses may have resulted in contamination of soil, vegetation, ground water, surface water or air. Any remaining or abandoned buildings and subsurface infrastructure on site may also still contain hazardous substances such as heavy metals and asbestos or be subject to fires and fly-tipping affecting the contaminant profile at the site. Contaminants from brownfield land may migrate on-site and off-site and can be released during remediation works. The exposure to harmful contaminants can occur via numerous pathways, including inhalation of vapours, or dust emitted from the site and ingestion of groundwater contaminated by the site [7].

There has been increasing interest in brownfield-related research. A large proportion of studies focused on the remediation process, economic impact, and risk assessment. Health impact assessments of brownfield land have been conducted in several locations [8–12], which focused on estimating potential population health effects due to proposed policy changes or intervention [13,14]. Yet, there is still a lack of epidemiological evidence that assesses the relationships between brownfield land and adverse health effects.

This study aims to systematically review the literature and synthesise the existing empirical evidence on associations between populations living near or on brownfield land and health relevant outcomes. Because there is no universal definition of brownfield land, we refer to the term as previously developed land that has subsequently become vacant or derelict. Brownfield land is potentially, but not necessarily, contaminated.

## Methods

### Search strategy

This systematic review on brownfield land and health was carried out according to the Preferred Reporting Items for Systematic Reviews and Meta-Analyses (PRISMA) reporting guidelines. The study protocol was registered on PROSPERO (CRD42022286826). We conducted our searches using EMBASE, MEDLINE, Global Health, Web of Science, Scopus and GreenFile. The searches included MeSH terms and were limited to human participants. We combined the keywords “brownfield” and its interchangeable terms such as “previously developed land”, and potential health outcomes such as “respiratory diseases” and “mortality”. We also used snowballing, which involves screening the reference lists of the included articles. We included studies from 1 January 1990 to 26 September 2022. The full search strategy is outlined in S1 Table.

### Eligibility criteria

Studies were eligible for inclusion if they met the following criteria: (1) Study type: Observational studies, including ecological, cross-sectional, case-control and cohort studies, published in peer-reviewed journals in English; (2) Population: No restrictions were applied to population characteristics; (3) Exposure: Brownfield land does not have a universal definition but is generally referred to as land or premises that has been previously used for industrial or commercial purposes but has subsequently become vacant, derelict, or contaminated. Therefore, we included the term “brownfield” and other similar terms including “previously developed land”, “derelict site” and “contaminated land”. Terms that are potentially linked to the previous use or contaminants of brownfield land were also included (e.g. petrol station); (4) Outcome: Due to limited research in this area, we did not confine the type of health outcome to be included in the review. Studies including at least one health outcome, biological markers of exposure or effect were also included. The outcomes could be self-reported, diagnosed, from routinely collected health and clinical records or death and birth registries. We excluded studies with an explicit focus on agricultural brownfield and farming as this was outside the scope of this review.

After the removal of duplicates, the search results were assessed independently by two authors (W.W. and D.F.) using the online tool Covidence. The screening started with the title and abstract screening and was followed by full-text screening against the eligibility criteria. Discrepancies highlighted by Covidence were resolved by a discussion between the two authors.

### Data extraction

Data extraction included information on authors, year of publication, title of the paper, study location, study design, study population, age groups, source and pathway of exposure, health outcome, measures of effects and effect size, covariates and significant findings. The information was extracted by the first author (W.W.) and checked by the last author (D.F.).

### Quality assessment

Quality assessment was conducted using a modified version of the Newcastle-Ottawa scale adapted for observational studies [15,16]. The scale consists of three criteria focusing on the selection (maximum 7 stars), comparability (maximum 2 stars) and outcomes (maximum 3 stars), with a maximum of 12 stars. A score of 0–4 was defined as poor quality, 5–8 as fair quality, and 9–12 as good quality.

### Data analysis

Due to the heterogeneity in exposure metrics, health outcomes and analytical methods between studies, the data could not be pooled and a meta-analysis was not performed. We instead conducted a narrative synthesis following the guidance by Popay et al. [17] in which studies were tabulated and categorised by exposure measure and outcome, and results were synthesised and structured into categories. Due to the small number of included studies, studies were assessed individually and the links between studies were identified.

## Results

The PRISMA flow diagram (S1 Fig) outlines the process of the literature search on brownfield land and potential health impacts and consequent screening. Our search initially identified 2,028 studies, of which 1,527 were unique. After title and abstract screening, 38 studies were assessed at the full-text screening stage. Six studies met the pre-defined eligibility criteria and were included in this review. The main reason for the full-text exclusion was due to study design (e.g. health risk assessment), publication type (e.g. abstracts and news articles) and exposures not related to brownfield land. We identified one study from the reference of a systematic review paper, however, this was not peer-reviewed and, therefore, not included in our review.

### Study characteristics

Key characteristics of the studies included in the review are reported in Table 1. Four studies reported results from the U.S. and two from the UK. The two UK studies were conducted by the same leading author, using the same exposure data and health outcomes, but were both included as they were analysed at different geographic units [18,19]. Similarly, two U.S. studies by the same leading author and using the same exposure data were included as they assessed different health outcomes [20,21]. Three of the six included studies used area-level health outcomes and exposures, and therefore, were classified as ecological [18,19,22]. Two were individual-level cross-sectional studies, with one based on a large population (*n* > 100,000) [23], and one based on a small population (*n* = 262) [20]. One longitudinal study collected serum samples from 774 participants throughout the study follow-up period, and 1,309 serum metal samples were included (45% of the participants had more than one measurement) [21].

**Table 1:** Characteristics of studies included in the systematic review.

First Author, Year	Study Design	Study Domain	Sample Size	Age (Years)	Brownfield Exposure	Health Outcomes	Confounders	Main Findings	Quality Score
Bambra, 2014 [19]	Ecological study, small-area analysis	England, UK	Not available	All	Each ward was assigned a relative measure of brownfield land (previously developed land (PDL)) based on individual sites within each ward and the percentage area of PDL within the ward.	1. Age-sex standardised **premature mortality** ratio 2. Age-sex standardised morbidity ratio for self-reported **general health** ’not good’ 3. Age-sex standardised morbidity ratio for self-reported general health ’limiting long-term illness’	Multiple environmental deprivation (Med-Ix), Townsend Index of Deprivation, ethnicity, education, unemployment, socioeconomic status, car/van owned, housing tenure, urban-rural classification	People with morbidity (’not good health’ and ’limiting long-term illness’) living in wards with a high proportion of brownfield land were significantly more likely to suffer from poorer health than those living in wards with a small proportion of brownfield land. Positive, yet statistically non-significant association with premature mortality.	11
Bambra, 2015 [18]					Further to the above, each ward was assigned to one of the nine regions in England.			Within each region, wards with large amounts of brownfield land were associated with higher premature mortality rate and poorer general health, with the exception of London which had the opposite direction of effect. North West had the highest amount of premature mortality rate and poorer general health cases compared to the other regions.	11
Litt, 2002 [22]	Ecological study, small-area analysis	Southeast Baltimore, Maryland, USA	Not available	**≥**45	Each census tract was assigned a score based on brownfield land characteristics including substance scores, years of operation and acreage, and then each tract was assigned to one of three zones based on hazard potential.	**Mortality** (endpoints included leading cause of death index, cancer (all-cause, lung, colon, bladder, stomach, oral, head and neck, skin), heart disease, COPD, diabetes, cerebrovascular disease, influenza and pneumonia, and liver disease).	Population age, area of census tract, percent owner-occupied homes, poverty status, minority populations, represented percent working class and educational attainment	Communities living in areas with ’high hazard potential’ brownfield land experienced higher mortality rate due to ‘top causes of death’ odds ratio(OR) = 1.20 (95% CI 1.10,1.31), cancer OR = 1.27 (95% CI 1.09,1.48), lung cancer mortality OR = 1.33 (95 CI% 1.03,1.73) and ‘respiratory mortality index’ (COPD, influenza, lung cancer) OR = 1.39 (95% CI 1.15,1.68), and COPD OR = 1.45 (95%CI 0.98, 2.12).	10
Lodge, 2020 [20]	Cross-sectional study	Detroit, Michigan, USA	262	**≥**18	Based on site names, brownfield sites were manually coded as industrial, gas station, auto shop, commercial, municipal, auto wash and unknown. Exposure to brownfields was coded “close” if the participant’s household was ≤200 m from the nearest brownfield (n = 66; reference) and “far” if > 200 m (n = 196).	Three **biomarkers**: 1. sjTRECs 2. C-reactive protein (CRP) 3. interleukin-6 (IL-6) (1 is a marker of naive T-cell production and ageing of the thymus; 2 and 3 are markers of systemic inflammation)	Age, gender, race/ethnicity, income, educational attainment, BMI, cigarette smoking status	Individuals living near brownfield sites had significantly lower naive T-cell production, suggesting accelerated immune ageing. Positive, yet statistically non-significant associations for the other biomarkers.	10
Lodge, 2022 [21]	Longitudinal study	Detroit, Michigan, USA	774	**≥**18	Brownfield exposure is presented as the distance-weighted brownfield density within 200 m of residence. The sites are assessed jointly (i.e. all sites) and separately, for sites meeting qualifications for environmental remediation under Part 201 ("Environmental Remediation", primarily industrial) and 213 ("Leaking Underground Storage Tank")	**Serum levels of heavy metals**: lead (Pb), mercury (Hg), manganese (Mn), and copper (Cu). Participants with serum collected during waves 1 (2008–09), 2 (2009–10), 4 (2011–12) or 5 (2012–13).	Age, gender, race/ethnicity, income, educational attainment, and census block group data on the percentage of households living at or below the federal poverty limit	Statistically significant and positive estimate of effect between increased density of brownfields listed under Part 201 (primarily industrial) and serum Hg. Serum Hg was also positively associated with Part 213 brownfields, but negatively associated with all-brownfield (statistically non-significant). Positive associations were found between serum Pb and all brownfield categories. No evidence for Mn and Cu (estimates of effect clustered tightly around the null for the different brownfield categories).	10
Slawsky, 2022 [23]	Cross-sectional study	North Carolina, USA	39,495	Neo-nates	Exposure metrics: Sum of the number of brownfields within 2,000 m of the residential address at birth. Brownfield exposure was broken into three bands: zero brownfields in 2,000 m (reference), one to five brownfields in 2,000 m, and more than six brownfields within 2,000 m.	**Birth defects**: 7 defect groups (central nervous, cardiovascular, orofacial, digestive, external, urinary, and chromosomal), including 30 individual phenotypes in total	Gestational parent age at delivery, race/ethnicity, prenatal care, gestational parent smoking and diabetes status, census block group urbanicity, areal level education, and Environmental Quality Index	Statistically significant and positive associations between any brownfield within 2,000 m and cardiovascular (OR = 1.07, 95% CI: 1.02,1.13) and external defect group (OR = 1.17, 95% CI: 1.01,1.35). Positive association between any brownfield and chromosomal defects group (OR = 1.05, 95% CI:0.96,1.15). Negative association between any brownfield and central nervous (OR = 0.98, 95% CI: 0.87,1.11), and digestive defects groups (OR = 0.97, 95% CI: 0.88, 1.06). All individual phenotypes had positive association with any brownfield; while the relationship is not uniform across phenotypes under the other defect groups. Compared to exposure group with zero brownfields, high density brownfield exposure group is associated with increased odds for various birth defects, except for digestive defects group.	11

**Abbreviations:** SE: Standard error; OR, odds ratio; COPD, chronic obstructive pulmonary disease; sjTRECs, Signal joint T-cell receptor excision circles.

Health outcomes that were analysed were (self-reported) general health, birth defects and mortality. Exposure and response biomarkers were also considered, where available. Four studies reported one type of outcome [20–23] and the two UK studies reported two health outcomes [18,19]. All studies accounted for age, socioeconomic and demographic characteristics. Other study-specific confounders included sex [18–21], environmental index [18,19,23], urban-rural classification [18,19], body mass index (BMI), smoking status [20] and area of census tract [22]. Studies used either linear mixed models [18,19], generalised linear regression models [20], linear generalised estimating equations [21], mixed effects logistic regression models [23] or log-linear models [22].

All studies assessed exposure to brownfield land via governments’ environmental inventory databases. There was no, or limited information regarding the previous use of the brownfield land that could indicate potential contamination. The three small-area studies gathered information on brownfield land (e.g. location and site classification), which was used to represent the level of exposure for each unit of area [18,19,22]. Two studies measured the distance between a participant’s home address to the nearest brownfield land as a proxy for brownfield exposure [20,21]. The longitudinal study created buffers around individual’s residence and categorised brownfield exposure into three bands based on the number of brownfield sites within each buffer [23].

### Methodological quality assessment

The quality score of the studies is summarised in Table 1 and the full assessment details are presented in S2 and S3 Tables. Overall, all studies had a clear research question and employed robust statistical methods. The exposure assessment method varied across studies, even for studies conducted in the same country. This may introduce detection bias in terms of the measurement or classification of exposure. The selected studies were of high quality with scores of 10–11 out of the maximum 12. The deducted scores were due to the use of self-reported health data [18,19], low representativeness of the exposed population [20–22], not controlling for age and sex [22,23], and/or small sample size [20]. However, it should be noted that although the assessment scale has been adapted for observational studies, there is currently no agreed checklist for ecological studies. Therefore, the quality scores for some studies may be overestimated.

### Summary of findings

We synthesised findings separately based on whether the study used an area-level or an individual-level exposure assessment. Within each of these types of exposure, studies were further stratified by health relevant outcomes: self-reported general health, mortality, birth defects and biomarkers. Most outcomes were only included in a single study.

#### Area-level exposure

Three studies conducted analyses at census area level [18,19,22]. Each census area was categorised into three bands, which were used as a proxy for the level of brownfield exposure. Briefly, Bambra et al. calculated a relative measure of brownfield land for each census area using the number of sites and the percentage area of brownfield land within the area, which were then grouped into areas with small, medium and high amounts of brownfield sites, respectively [18,19]. Litt et al. developed brownfield scoring algorithms based on site characteristics and potential substances on site. Site scores within each census area were aggregated to obtain an area score, which categorised areas into high, medium and low “hazard potential” [22]. Two types of health outcomes, mortality and general health, were explored using the area-level exposures.

#### Self-reported general health

Bambra et al. assessed general health in two studies, using self-reported health data from the 2001 UK Census [18,19]. For each area, the authors calculated the proportions of people self-reporting their general health as ‘not good’ and having ‘limiting long-term illness’. The health outcomes were age- and sex-standardised, and both studies included socioeconomic status (SES), multiple environmental deprivation and demographic characteristics as confounders. The first study [19] reported that the average rate of people suffering from poorer general health was 14.3–15.4% higher in areas with a large proportion of brownfield land, compared to those living in areas with a small proportion of brownfield land. The rate was lower for the population living in areas with a medium proportion of brownfield land (5.4–8.7%). The second study [18], which assessed within and between region effects, also found the highest association in the North West, with an excess of 27.5%, 23.0% and 20.2% ‘not good health’ in large, medium and small areas, respectively, compared to the reference region South East. The rate was slightly lower for ‘limiting long-term illness’, with an excess of 18.2%, 15.6% and 15.1% in large, medium and small areas, respectively. Within the North West, there were an additional 3.3% of people with ‘limiting long-term illness’ and 6.7% self-reporting their health as ‘not good’ in areas with a large proportion of brownfield land compared to those with a small proportion of brownfield land.

#### Mortality

All-cause premature mortality and leading causes of mortality were explored in all three studies [18,19,22]. Bambra et al. conducted two UK studies at the ward level. Electoral ward is a key administrative division in England. There are over 7,000 wards and the average population in each ward is around 5,500 [24]. The first study found a statistically significant, association between quantity of brownfield land and mortality, however, the associations were statistically non-significant after adjustment for SES, multiple environmental deprivation and demographic characteristics [19]. A follow-up study [18] used the same exposure data at ward level but focused on assessing if the associations varied within and between the nine regions of England (a sub-national division). They observed that brownfield land was regionally patterned in England, with higher density in the Northern regions. Using the South East as a reference, the North West had 12.5%, 10.0% and 7.6% adjusted higher all-cause premature mortality rate in large, medium and small brownfield land areas, respectively. Within the North West, the authors reported a 9.4% increase in premature mortality rate in wards with a large compared to a small percentage of brownfield land.

Litt et al. evaluated brownfield land in Southeast Baltimore, U.S. at the census tract level, which is a statistical subdivision of a county or statistically equivalent entity in the U.S. [22]. Southeast Baltimore has 28 census tracts, and each represents approximately 4,000 people. The study reported that people aged over 45 years living in “high hazard potential areas”, when compared with the same age group living in “low hazard potential areas”, experienced higher mortality rate due to cancer (odds ratio (OR): 1.27; 95% confidence interval (CI): 1.09,1.48), lung cancer (OR: 1.33; 95 CI%: 1.03–1.73) and a “leading causes of death” index (index of liver, diabetes, stroke, COPD, heart diseases, cancer, injury, and influenza and pneumonia; OR: 1.20; 95% CI: 1.10,1.31). The models were adjusted for age and area of census tract, and the associations remained significant after adjusting for SES. The study also found significant associations with respiratory mortality index (including COPD, influenza and lung cancer; OR: 1.39; 95% CI: 1.15,1.68), but not COPD mortality alone (*p* > 0.05). The study found no effect on the population living in the medium hazard potential areas with any outcome.

#### Individual-level exposure

Three studies assessed exposure at the individual level. The studies used proximity to and/or density of brownfield land as the proxy for brownfield exposure. Lodge et al. calculated the distance between individual’s residential address and the nearest brownfield site. The exposure to brownfield land was coded as “close” (≤ 200 m, reference) and “far” (> 200 m), representing groups with relatively high and low exposure, respectively [20,21]. Slawsky et al. summed the number of brownfield sites within 2,000 m of the residential address at birth [23]. They also categorised brownfield density into three bands: zero brownfield sites in 2,000 m (reference), one to five brownfield sites in 2,000 m, and more than five brownfield sites within 2,000 m [23]. Using individual-level exposures, the studies explored two types of health relevant outcomes, birth defects and biomarkers of exposures (inflammatory mediators) and response (serum metals).

#### Birth defects

Using North Carolina birth records from 2003–2015, Slawsky et al. analysed seven birth defect groups (central nervous, cardiovascular, orofacial, digestive, external, urinary and chromosomal) and 30 distinct phenotypes under the groups [23]. The study found significant associations (all adjusted for gestational parent age, smoking and diabetes status, prenatal care, race/ethnicity, socioeconomic status (SES) and environmental covariates) between any brownfield site within 2,000 m and cardiovascular (OR: 1.07; 95% CI: 1.02,1.13) and external defect group (OR: 1.17; 95% CI: 1.01,1.35). Individual phenotypes were mostly null, except for positive associations observed with atrial septal defect (OR: 1.08; 95% CI: 1.01,1.16) and ventricular septal defect (OR: 1.15; 95% CI: 1.03,1.28), and an inverse association with gastroschisis (OR: 0.74; 95% CI: 0.58,0.94). For the multi-band brownfield exposure, high brownfield exposure (more than six sites within a 2,000 m buffer) showed significant associations with cardiovascular (OR: 1.25; 95% CI: 1.13,1.39) and urinary group (OR: 1.19; 95% CI: 1.02,1.38), compared to no brownfield sites within 2,000 m. Most individual defects showed null associations.

#### Biomarkers

Lodge et al. assessed the relationship between residential proximity to brownfield land and three biomarkers: sjTRECs (a marker of naive T-cell production and ageing of the thymus; fresh thymocyte cell-suspensions were analysed by flow cytometry using anti-CD4 FITC and anti-CD8 PE monoclonal antibodies [25]) and two markers of systematic inflammation, C-reactive protein (CRP) and interleukin-6 (IL-6) [20]. The study used the longitudinal observational cohort Detroit Neighbourhood Health Study. They reported a positive association between proximity to brownfield land (≤ 200 m) and a one-unit decrease in sjTRECs per million whole blood cells in adults (≥ 18 years), suggestive of accelerated immune ageing: 0.30 (95% CI: 0.59,0.02, *p* = 0.04; adjusted for age, gender, race, BMI, smoking status and SES). Decreased T-cell production associated with brownfield proximity may be caused by toxicant exposure in brownfield land, or may serve as a marker of other unresolved neighbourhood stressors that were not considered in the study [20,26].

Lodge et al. conducted another study using information from the same cohort to evaluate the effect of residential proximity to brownfield land on serum lead (^208^Pb), mercury (^202^Hg), manganese (^55^Mn), and copper (^63^Cu) [21]. Each serum sample was digested and diluted, and inductively-coupled plasma mass spectrometry (ICP-MS) procedures were used to quantify ^208^Pb, ^55^Mn, and ^63^Cu, and helium gas was used for the measurement of ^202^Hg. The study assessed brownfield exposure for all brownfield sites and separately for sites meeting qualifications for environmental remediation under ‘Environmental Remediation’ (primarily industrial sites) and ‘Leaking Underground Storage Tank’. All models were adjusted for age, gender, race/ethnicity and SES. Results only showed a significantly positive association for serum Hg, with one standard deviation increase in residential exposure to ‘Environmental Remediation’ brownfields within 200 m associated with a 0.06 (95% CI: 0.03,0.09) log_e_-unit increase in serum Hg level. Pb was the only serum metal that had consistently statistically non-significant positive estimates of effect for all brownfield sites (0.04, 95% CI: -0.01,0.09) and brownfield sites listed separately under ‘Environmental Remediation’ (0.04, 95% CI: -0.03,0.10) and ‘Leaking Underground Storage Tank’ (0.02, 95% CI: -0.00,0.04). No associations were found in the other metal serums. The study also tested residential proximity to each exposure within different distances (200 m, 400 m, and 800 m) but the effect estimates did not change.

## Discussion

### The health impact of brownfield land

We systematically reviewed the epidemiological evidence on the effect of brownfield land on the nearby population. Although outcomes varied, the included studies all found evidence for a potential link between living in close proximity to or in an area with a higher density of brownfield land and adverse health effects or biomarkers. Several studies mentioned the potential mechanisms (e.g. air, water and soil) for the health impacts, which can be summarised into physical contamination and psychological affections (e.g. odour, noise, aesthetic nuisance) [18,20,22,23]. Depending on the previous use, brownfield land may contain hazardous waste such as heavy metals (e.g. arsenic, lead and cadmium), chemical substances (e.g. solvents), oil, tar, gases (e.g. volatile organic compounds), asbestos and radioactive substances. The health impact of hazardous waste has been extensively researched [27–33], including the health outcomes that were outlined in our review. Some brownfield sites may be ex-storage or commercial buildings which tend not to generate contaminants or pollutants from their day-to-day operations, or sites that remain hard-standing, which lessens migration. However, studies show that vacant or derelict land is not just an eyesore but can affect well-being, such as negative emotions, heart rate variability and stress-led inflammatory responses [34–36]. In addition to mental health, some abandoned sites may be associated with anti-social behaviour or injury hazards, such as fire, fly-tipping and trip hazards, which could be linked to some of the health outcomes including general health, all-cause mortality and an increased rate of birth defects.

### Methodological considerations

Three out of six studies used an ecological study design and analysed individual data aggregated to census geographies. Such area-level analyses are based on the spatial differences in environmental and/or socioeconomic factors across areas, which are used to assess the relationship between these factors and health outcomes [37]. The study design is particularly useful in studies where individual-level exposure or health outcomes are not available [13]. However, ecological studies are prone to ecological fallacy and the findings at the area level may not hold at the individual level [38]. It is also possible that the associations would vary largely at different geographic levels. Most of the studies adopted an ecological or cross-sectional design, which may be linked to available data on brownfield exposure which is mostly from administrative sources with no or limited temporal information. Three studies assessed individual-level outcomes and used either residential proximity or density of brownfield land. Such analyses might be affected by the participants’ activity and mobility patterns. One study conducted a sensitivity analysis in which participants who moved house were excluded and found similar results to the main analysis [21]. Nevertheless, the information on activity patterns could help identify long/short-term exposure and reduce exposure misclassification.

The selected studies clustered population/areas into groups that represent different bands of exposure, in which the lowest band was used as a reference group. Because the studies have no or very limited information on site contamination, various approaches were adopted to evaluate the level of exposure. Litt et al. offered more insight into the potential hazardous exposure on each site, which then gave a more meaningful classification of exposure risk groups [22]. However, the information was obtained through consulting multiple resources, which may not suit large-scale projects. Lodge et al. investigated a random sample of the collected sites by manually categorising the sites into pre-defined facility types based on site names [20]. The categories include industrial, gas station, auto shop, commercial, municipal, auto wash and unknown. This dataset was used to perform a descriptive statistic on the brownfield sites but was not used in the health analysis. Furthermore, the selected studies included either mixed development status [18–21,23] (i.e. un-remediated/undeveloped or remediated/developed) or no status [22] of brownfield land. Ideally, sites of different development statuses should be assessed separately as they may be associated with different health outcomes and policy implications. Overall, although detailed information on brownfield land (e.g. previous use, site contamination) could aid a better understanding of the relationship of certain types of brownfields (e.g. industrial) on human health, we found the approaches were appropriate for the available information on brownfield land and participants in each study.

All studies included a wide range of socioeconomic and demographic characteristics as confounders in the analysis. However, there are other confounders (e.g. diet) that are difficult to control for. Litt et al. provided the distribution of brownfield sites along with spatial distribution of percentage minority, poverty status, less than high school degree, family income, home-owner occupancy and working class, but did not discuss the spatial linkage of these factors [22]. There is evidence that areas nearer to industrial sites tend to be more deprived [39]. This is likely due to the low costs of land and development associated with industrial development in more deprived areas, and the blight caused by industrial development may further depress land and housing values nearby [20]. The two UK studies explicitly focused on health inequalities in their study design and compared the estimated effects between areas [18,19]. It is well-documented that the physical environment is a strong determinant of health inequalities [18]. The inequality could be triggered by the uneven spatial distribution of brownfield sites (i.e. brownfield sites are more prevalent in certain areas) and/or brownfield sites with higher risks (both real and perceived) are located in more deprived areas. However, studies may be subjective to self-election bias as it remains unclear whether individuals with greater health needs are more likely to reside in areas with more brownfield land or individuals of lower SES and economic power are forced to live near brownfield sites due to such areas often being less desirable and consequently house prices being lower. Further research is required to address the issue of health inequality.

### Strengths and limitations

This is the first systematic review on brownfield land and health. The review synthesised evidence based on area-level and individual-level exposure and identified various health outcomes that have potential links to exposure to brownfield land. Although we followed a standardised protocol, our review has some limitations. As most of the studies were ecological and cross-sectional, which capture data at a specific point in time, there is a very limited evidence base for the temporal change of the exposures and their potential health impacts. In addition, we were not able to assess the health impacts on vulnerable sub-groups (except for neonates). Furthermore, the included studies did not have information on contaminants or previous use of the brownfield land. As highlighted above, it is preferred to have such information so that specific contaminants or functions (e.g. gas station, waste treatment facility) which may pose higher risks to the communities could be assessed separately. Due to the broad definition of brownfield land, it is difficult for studies to adopt a standardised method to assess exposure to brownfield land or consider all the exposure pathways (e.g. water, diet). As a result, all included studies are inevitably subject to exposure misclassification. The majority of studies used proximity to or density of brownfield land as a proxy for the exposure assessment. However, the threshold for proximity (200 to 2,000 m) or density varied by study, which made it not possible for this review to conduct a meta-analysis of the findings.

### Policy implication and future research

The redevelopment of brownfield land is an attractive solution to address the increasing housing demand, especially as the infrastructure (roads, services) is already present. Redeveloping brownfield land has great potential to help meet the sustainable development goals (SDGs) in “make cities and human settlements inclusive, safe, resilient and sustainable” [40]. This would involve creating sustainable cities and communities through the reuse of vacant and derelict land and preventing urban sprawl. To this end, several countries have established administratively collected brownfield land inventories. The policies around brownfield land have generally focused on planning and economic values, mostly the redevelopment for housing. For example, the Brownfield Land Registers in the UK require local planning authorities to detail site location, size, ownership, planning status and number of potential dwellings. However, from a prevention and public health planning perspective, an improved understanding of the potential health hazards associated with brownfields or specific past land uses may assist in the prioritisation of remedial activity or influence the development process of the land. When planning the redevelopment of brownfield sites, it is crucial to consider the potential impact on health and well-being of the surrounding communities, particularly those already experiencing health disparities. Efforts should be made to ensure that the redevelopment of brownfield sites incorporates measures to mitigate environmental risks and promote health equity. This can include thorough remediation of contaminated areas, implementing sustainable design practices, obtaining local communities’ perspectives, and considering their needs, which may ultimately contribute to the success of the projects [3]. In particular, where an association between certain previous use and health outcome has been established, the evidence should be implemented in the planning process.

This review highlights the lack of a universal definition of brownfield land, which also leads to mixed methodologies in quantifying brownfield exposures. Future studies that explore the health impacts on a subset of brownfield sites with certain contaminants (e.g. heavy metals) or previous use (e.g. quarry, gas works) would be beneficial. Additionally, although the term ‘brownfield’ has been used in publications in various countries and regions, such as China, Canada and the European Union, we only found epidemiological evidence from the UK and U.S. This limited the generalisability of the findings, as countries have different social, environmental, and policy barriers and enablers and the results may not be directly transferable. Therefore, there is a need for future research from diverse geographic regions to gain a more comprehensive understanding of the associated health outcomes.

## Conclusions

This systematic review synthesised the existing epidemiological evidence on the potential health impacts in populations exposed to brownfield land. The included studies are of high quality, using appropriate exposure assessment, outcome and robust statistical methods. Four types of health-relevant outcomes were included in the review: self-reported general health, mortality, birth defects and biomarkers (biomarkers of exposures (inflammatory mediators) and response (serum metals)). All studies found significant associations between at least one outcome and people living in closer proximity to brownfields or in areas with higher proportions of brownfield sites. However, the strength of this conclusion is limited, due to the paucity of health studies in this area and the absence of detailed exposure information. Further studies are required to contribute to the evidence bases to inform future housing policies and urban planning.

## Supporting information

S1 Checklist PRISMA 2020 checklist. (PDF)

S1 Fig Study selection flow chart. (PDF)

S1 Table Search strategy for brownfield and health on EMBASE. (PDF)

S2 Table Adapted Newcastle-Ottawa Scale used for quality assessment. (PDF)

S3 Table Quality assessment score using the adapted Newcastle-Ottawa Scale. (PDF)
