# Supplementary figures and images for: Brownfield land and health: A systematic review of the literature
Source: PLoS One. 2023 Aug 4;18(8):e0289470. doi: 10.1371/journal.pone.0289470 (PMC10403084; doi:10.1371/journal.pone.0289470)

**S1 Fig. Study selection flow chart.**

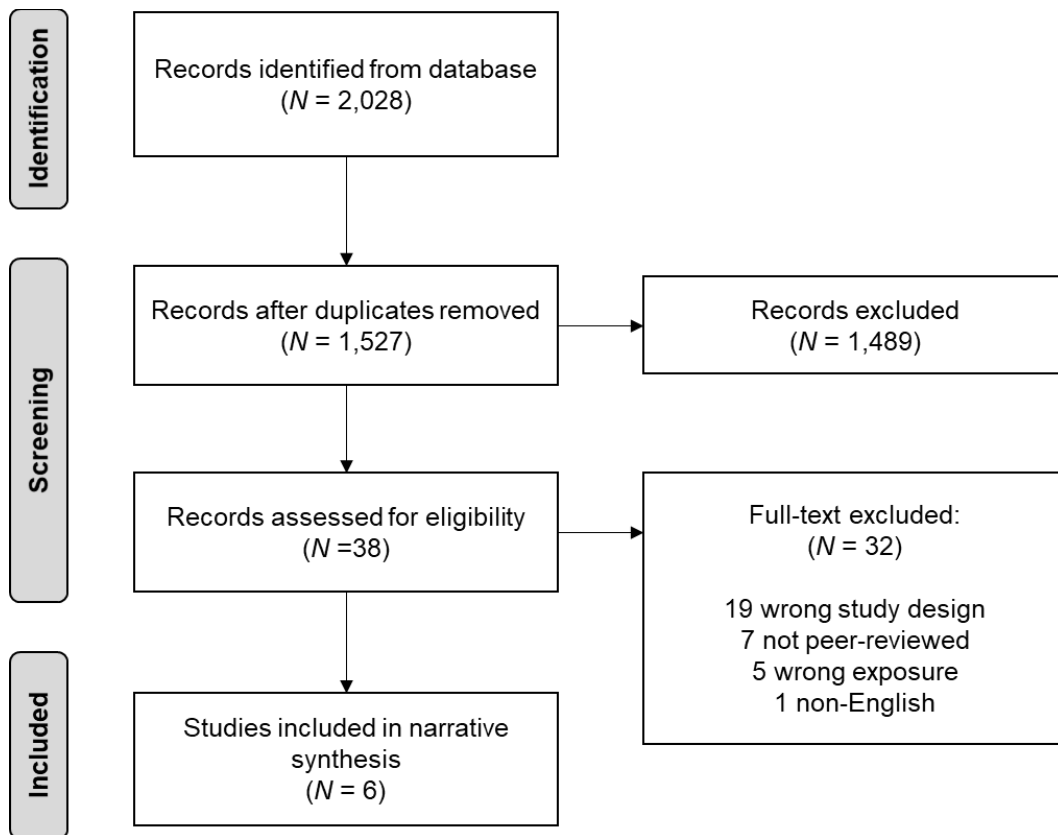

Supplement: S1 Fig — (PDF) [file pone.0289470.s002.pdf]
