# Supplementary material for: Brownfield land and health: A systematic review of the literature
Source: PLoS One. 2023 Aug 4;18(8):e0289470. doi: 10.1371/journal.pone.0289470 (PMC10403084; doi:10.1371/journal.pone.0289470)
Supplement: S1 Table — (PDF) [file pone.0289470.s003.pdf]

S1 Table. Search strategy for brownfield and health on EMBASE.

1	(participant* or individual*).mp.
2	social environment/ or community/ or community living/ or neighborhood/
3	exp population/
4	human/
5	(brownfield* or previously developed land* or derelict land*).mp.
6	land regeneration.mp.
7	land redevelopment.mp.
8	urban redevelopment.mp.
9	contaminated land.mp.
10	*industrial area/
11	(gas station or petrol station).mp.
12	exp waste disposal facility/
13	soil pollution/
14	heavy metal/
15	exp dangerous goods/
16	housing/
17	*death/
18	*mortality/
19	*morbidity/
20	exp respiratory tract disease/
21	exp cardiovascular disease/ or cvd.mp.
22	exp prematurity/
23	exp pregnancy outcome/
24	cancer.mp.
25	public health/
26	adverse event/
27	health care disparity/
28	biological marker/
29	exp agriculture/
30	exp animal/
31	1 or 2 or 3 or 4
32	5 or 6 or 7 or 8 or 9 or 10 or 11 or 12 or 13 or 14 or 15 or 16
33	17 or 18 or 19 or 20 or 21 or 22 or 23 or 24 or 25 or 26 or 27 or 28
34	31 and 32 and 33
35	34 not (29 or 30)
36	limit 35 to yr="1990 -Current"
