# Supplementary material for: Brownfield land and health: A systematic review of the literature
Source: PLoS One. 2023 Aug 4;18(8):e0289470. doi: 10.1371/journal.pone.0289470 (PMC10403084; doi:10.1371/journal.pone.0289470)
Supplement: S2 Table — (PDF) [file pone.0289470.s004.pdf]

S2 Table. Adapted Newcastle-Ottawa Scale used for quality assessment.

Selection	S1	Representativeness of the exposed population	Truly representative of the average in the target population (random sample or whole population)	**
			Somewhat representative of the average in the target population (purposive sampling of the target population)	*
			Selected group of population (e.g. only certain socio-economic groups) OR no description of the study population	
	S2	Selection of the non-exposed/less-exposed population	Drawn from the same community as the exposed	*
			Drawn from a different community as the exposed population OR no description of the derivation of the non-exposed/less-exposed population	
	S3	Ascertainment of the exposure	Records of brownfield sites from reliable data sources, records include detailed information on characteristics of sites (e.g. location, size, previous use,)	**
			Records of brownfield sites from reliable data sources, with limited (e.g. location only) or no information on characteristics of sites	*
			Data sources not reliable OR no description	
	S4	Sample size	Justified and satisfactory	**
			Not justified or small sample size (<1000)	
Comparability	C1	The subjects in different outcome groups are comparable, based on the study design or analysis. Confounding factors are controlled	The study controls for age and sex (the most important factor)	*
			The study controls for any additional factors (e.g. socioeconomic status, region)	*
Outcome	O1	Assessment of the outcome	Independent blind assessment.	**
			Record linkage (e.g. identified through ICD codes on database records)	**
			Self report	*
			No description	
	O2	Statistical test	The statistical test used to analyse the data is clearly described and appropriate, and the measurement of the association is presented as either an odds ratio, confidence interval and p-value or a beta coefficient, standard error and p-value	*
			Not described, or incomplete	
