# Supplementary material for: Brownfield land and health: A systematic review of the literature
Source: PLoS One. 2023 Aug 4;18(8):e0289470. doi: 10.1371/journal.pone.0289470 (PMC10403084; doi:10.1371/journal.pone.0289470)
Supplement: S3 Table — (PDF) [file pone.0289470.s005.pdf]

S3 Table. Quality assessment score using the adapted Newcastle-Ottawa Scale.

Author, year	S1	S2	S3	S4	C1	O1	O2	Score
Bambra et al., 2014	**	*	**	**	**	*	*	11
Bambra et al., 2015	**	*	**	**	**	*	*	11
Litt et al., 2002	*	*	**	**	*	**	*	10
Lodge et al., 2020	*	*	**	*	**	**	*	10
Lodge et al., 2022	*	*	**	**	**	**	*	11
Slawsky et al., 2022	**	*	**	**	*	**	*	11
